# CHI3L1 in the CSF is a potential biomarker for anti-leucine-rich glioma inactivated 1 encephalitis

**DOI:** 10.3389/fimmu.2022.1071219

**Published:** 2023-01-05

**Authors:** Jinyi Li, Hongyan Li, Yunhuan Wang, Xiuhe Zhao, Shengjun Wang, Ling Li

**Affiliations:** ^1^ Department of Neurology, Qilu Hospital, Cheeloo College of Medicine, Shandong University, Jinan, Shandong, China; ^2^ Department of Neurology, Qilu Hospital, Cheeloo College of Medicine, Shandong University, Qingdao, Shandong, China

**Keywords:** LGI1 encephalitis, cerebrospinal fluid, chitinase 3-like 1, modified rankin scale, prognosis

## Abstract

**Objective:**

Anti-leucine-rich glioma inactivated 1(LGI1) encephalitis is one rare autoimmune encephalitis which is accompanied by inflammatory responses. (Anti-leucine-rich glioma inactivated 1 (anti-LGI1) encephalitis is an autoimmune disease mediated by inflammatory responses.)This study aimed to investigate the Chitinase 3-like 1(CHI3L1) in anti-LGI1encephalitis patients and evaluate its association with modified Rankin Scale (mRS) score in anti-LGI1 encephalitis at admission and 6 months follow-up.(This study looked into the relationship between Chitinase 3-like 1 (CHI3L1) and the modified Ranking Scale (mRS) score in anti-LGI1 encephalitis patients at admission and 6 months later.)

**Methods:**

Thirty-five patients with anti-LGI1 encephalitis and 22 patients with non-inflammatory neurological disease were enrolled in this study. (We enrolled 35 patients with anti-LGI1 encephalitis and 22 patients with non-inflammatory neurological disease.)Cerebrospinal fluid (CSF) and serum levels of CHI3L1 were measured by enzyme-linked immunosorbent assay. (We quantified CHI3L1 in the serum and cerebrospinal fluid (CSF) by performing an enzyme-linked immunosorbent assay.)Patients were evaluated for mRS score at admission and at 6 months follow-up.(We recorded the mRS score of the patients at admission and 6 months later.)

**Results:**

CHI3L1 levels in CSF and serum were highly elevated in patients with anti-LGI1 encephalitis at admission compared those with the controls.(At admission, patients with anti-LGI1 encephalitis had elevated CHI3L1 levels in the CSF and serum.) Additionally, patients presenting with cognitive impairment had significantly higher CSF CHI3L1 levels and mRS scores than those without cognitive impairment symptoms. Patients presenting with only faciobrachial dystonic seizures at admission had lower CSF CHI3L1 levels than those with other symptoms. Finally, CSF CHI3L1 levels were positively correlated with CSF lactate levels.

**Conclusion:**

CHI3L1 level in CSF is correlated with the severity and prognosis of anti-LGI1 encephalitis. (CSF CHI3L1 levels are correlated with the severity and prognosis of anti-LGI1 encephalitis.)

## Introduction

Anti-leucine-rich glioma inactivated 1 (anti-LGI1)encephalitis is a type of rare autoimmune encephalitis (1). The main clinical manifestations of anti-LGI1 encephalitis are memory loss, seizures, mental behavioral abnormalities and faciobrachial dystonic seizures(FBDS) (2–4). Chitinase-3 like-protein-1 (CHI3L1), a chitinase-like protein, is generated and released by various cells, including macrophages, microglia, including macrophages, microglia, neutrophils, synoviocytes, chondrocytes, fibroblast-like cells, smooth muscle cells, and tumor cells (5–8). CHI3L1 forms a multimeric complex with interleukin-13 receptor α2 and interacts with transmembrane protein 219 (TMEM219), activating the Erk, Akt, and Wnt-linked signaling pathways and suppressing inflammatory cell apoptosis (5, 9, 10). Some studies have shown that patients with anti-N-methyl-d-aspartate receptor (NMDAR) encephalitis have higher CHI3L1 levels in the cerebrospinal fluid (CSF) than viral encephalitis patients or healthy people (11, 12). The study showed thatCSF CHI3L1 levels were positively correlated with the modified Rankin Scale (mRS) score and serum interleukin-6(IL-6) in anti-NMDAR encephalitis (12), suggesting that CSF CHI3L1 level may be positively correlated with the severity of anti-NMDAR encephalitis. However, few studies have documented serum and CSF CHI3L1 levels in patients with anti-LGI1 encephalitis. Thus, we investigated the changes in serum and CSF CHI3L1 levels in anti-LGI1 encephalitis patients and their relationship with severity and prognosis. In parallel, this study analyzed the association between clinical characteristics and mRS score at admission and 6 months later.

## Material and methods

### Patients and controls

We retrospectively studied 35 patients with anti-LGI1 encephalitis. All included patients met the diagnostic criteria for anti-LGI1 encephalitis (13). The control group comprised 22 noninflammatory neurological disorder patients, which included migraine (n = 5), anxiety disorder (n=8), cervical/lumbar disc herniation (n=5), ischemic cerebrovascular disease (n=4). All patients underwent a lumbar puncturewithin 7 days after admission for a cerebrospinal fluidCSF examinationanalysis before starting their immunosuppressants treatment. (**Table 1**)We assessed the patients’ neurological status by recording their mRS score (14) at admission and 6 months after discharge.

**Table 1:** Clinical data and laboratory findings of anti-LGI1 encephalitis patients (n=35)and controls (n=22).

	Anti-LGI1 encephalitis	Control
Age of onset (years, mean ± SD)	61.91 ± 11.20	59.70 ± 11.73
male/Female	27/8	13/9
CSF samples(%)	30/35(85.71)	22/22 (100)
Serum samples(%)	30/35(85.71)	22/22 (100)
Clinical features(%)		
Psychiatric/behavioral problems	14/35(40.00)	
cognitive disturbance	22/35(62.86)	
Seizures	14/35(40.00)	
FBDS	20/35(57.14)	
Initial mRS (median,IQR)	2 (1–3)	
6-months follow-up mRS (median, IQR)	1 (1–2)	
Abnormal EEG(%)	23/33(69.70)	
Abnormal brain MRI(%)	15/34(44.12)	
CSF WBC count (×106, median, range)	1.6 (1–4)	1 (1–2)
CSF Protein (g/L, median, IQR)	0.38(0.27-0.50)	0.40 ± 0.18
Anti-LGI1 antibodies in CSF(%)	34/35(97.14)	
Anti-LGI1 antibodies in Serum(%)	32/33(96.97)	
Hyponatremia(%)	20/35(57.14)	
CSF CHI3L1 (pg/ml)	1216.30(511.78-1750.24)	493.93(354.90-735.98)
Serum CHI3L1 (pg/ml)	919.86(600.09-3110.75)	610.01(388.66-1355.32)

CSF, Cerebrospinal fluid; FBDS, faciobrachial dystonic seizures; mRS, modified Rankin Scale scores; IQR, Inter quartile range; EEG, Electroencephalogram; MRI, Magnetic Resonance Imaging; WBC, white blood cell; CHI3L1, Chitinase 3-like 1.

The local ethics committee at the Qilu Hospital of Shandong University approved this study, and all participants provided written informed consent.

### Quantification of serum and CSF CHI3L1

CSF and serum samples were centrifuged immediately after collection and then stored at −80°C for testing. Commercially available sandwich ELISA kits were used according to the manufacturer’s instructions to quantify CHI3L1 in the CSF and serum. (Elabscience Biotechnology Co. Ltd).

### Statistical analysis

All statistical analyses were performed using SPSS 26. Continuous variables of normally distributed data were presented as the mean ± standard deviation. Non-normally distributed datawere presented as the median and interquartile range (IQR). Groups were compared using Student’s t-test. Since CHI3L1 concentrations are non-normally distributed, we compared the anti-LGI1 encephalitis group and the control group using the Mann Whitney U. Spearman’s test was used to assess the correlation between CSF CHI3L1 levels and mRS scores. Correlations between the CHI3L1 levels and the clinical data were analyzed by multiple linear regression. Receiver operator characteristic (ROC) curves were used to assess the discriminating power of CHI3L1 levels in anti-LGI1 encephalitis patients. A value of *p* < 0.05 was considered statistically significant.

## Results

### Clinical features and demographics

**Table 1** lists the clinical features of the included patients. The mean age of the patients with anti-LGI1 encephalitis was 61.91 ± 11.20 years, and that of the control group was 59.70 ± 11.73 years. The male to female ratio of patients recruited with anti-LGI1 encephalitis versus controls was 27/8 and 13/9 respectively. The median mRS score during the onset of anti-LGI1 encephalitis patients was 2 (1–3). The median mRS score during the 6 months follow-up of anti-LGI1 encephalitis patients was1 (1–2). All anti-LGI1 encephalitis patients were treated with methylprednisolone alone or combined with intravenous immunoglobulin during phase of disease, followed by a gradual reduction of the prednisone dose.

### Clinical features related to mRS at admission and 6 months later in anti-LGI1 encephalitis patients

A Simple linear regression analysis revealed that the mRS score at admission was positively associated with hyponatremia(β=0.468, p=0.003) and negatively associated with FBDS (β=-0.426, *p*=0.005). The multiple linear regression analysis also revealed that the mRS score at admission was positively associated with hyponatremia(β=0.364, *p*=0.025) and negatively correlated with FBDS (β=-0.395, *p*=0.016). The mRS score at admission was not associated with serum/CSF LGI1 antibody titers, abnormal brain MRI, CSF protein concentrations, CSF white blood cell count, CSF immunoglobulin G, A, and M titers, and CSF lactate levels.

Meanwhile, the simple linear regression analysis and multiple linear regression analysis revealed that mRS score at the 6-month follow-up examination was not associated with serum/CSF LGI1 antibody titers, abnormal brain MRI, hyponatremia, FBDS, CSF protein concentrations, CSF white blood cell count, CSF immunoglobulin G, A, and M titers, and CSF lactate levels.

### Increased CSF and serum CHI3L1 levels in anti-LGI1 encephalitis patients

**Figure 1:**
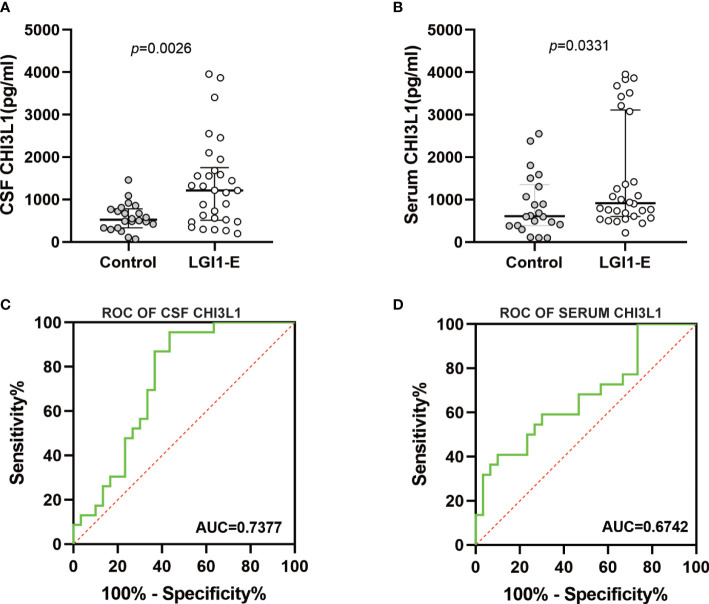
Levels of CHI3L1 in cerebrospinal fluid (CSF) and serum. CSF **(A)** and serum **(B)** CHI3L1 levels in patients with anti LGI1 encephalitis and controls;**(C)**, **(D)**Receiver operating characteristic curves for CSF and serum CHI3L1 to discriminate anti-LGI1 encephalitis patients from control patients.

We quantified CHI3L1 in CSF (*n* =30) and serum samples (*n* =30) from patients with anti-LGI1 encephalitis (*n* =35), and controls (*n* = 22) using an ELISA assay. Patients with anti-LGI1 encephalitis had significantly higher CHI3L1 levels in the serum and CSF than controls. (*p*=0.0026 and *p*=0.0331, respectively; **Figures 1A, B**).

Next, we evaluated whether CHI3L1 levels could be used to identify anti-LGI1 encephalitis patients using ROC curves. The area under the ROC curve (AUC) of CSF and serum CHI3L1 levels were 0.7377 and 0.6742, respectively (**Figures 1C, D**). The optimal cut-off values for CSF and serum levels were 868.6 pg/mL and 712.12pg/mL, respectively.

### Anti-LGI1 encephalitis patients with cognitive impairment symptoms had high CSF levels and mRS scores

**Figure 2:**
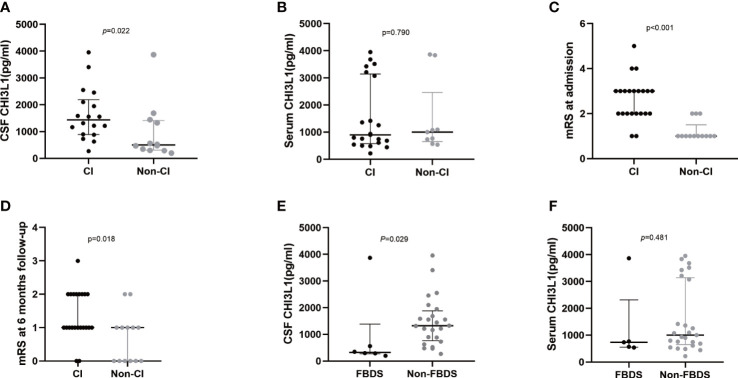
CHI3L1 level difference in serum and cerebrospinal fluid of LGI1 patients with different clinical presentations. Cerebrospinal fluid **(A)** and serum **(B)** CHI3L1 levels in anti-LGI1 encephalitis patients with and without cognitive impairment; mRS scores at admission **(C)** and at 6-month follow-up **(D)** of anti-LGI1 antibody encephalitis presenting with cognitive impairment and those without cognitive impairment symptomes; Cerebrospinal fluid **(E)** and serum **(F)** CHI3L1 levels in anti-LGI1 encephalitis patients presenting with only FBDS and other symptoms; CI: Patients presenting with cognitive impairments, Non-CI: Patients presenting without cognitive impairments.

Patients with anti-LGI1 encephalitis presenting with cognitive impairments (n = 18) had significantly higher CSF CHI3L1 levels than those without cognitive impairment symptoms (n = 12, p = 0.022, **Figure 2A**), but these two groups had similar serum CHI3L1 levels (**Figure 2B**). Patients with anti-LGI1 encephalitis who presented with cognitive impairments had significantly higher mRS scores on admission (p < 0.001, **Figure 2C**) and at the 6-month follow-up examination (p = 0.018, **Figure 2D**) than those without cognitive impairment symptoms.

Next, patients with other symptoms, such as psychotic behavior abnormalities or seizures, and those without these symptoms had similar CHI3L1 levels. Interestingly, anti-LGI1 encephalitis patients who presented with only FBDS at admission (n = 6) had significantly lower CSF CHI3L1 levels than those without FBDS symptoms (n = 24, p = 0.029; **Figure 2E**). However, these two groups had similar serum CHI3L1 levels (p = 0.481; **Figure 2F**).

### Clinical features related to increased CSF CHI3L1 concentrations in anti-LGI1 encephalitis patients

**Figure 3:**
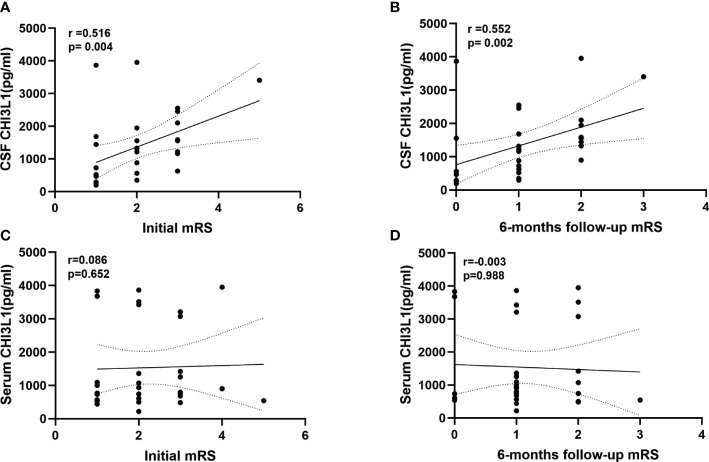
Levels of CHI3L1in cerebrospinal fluid (CSF) and serum association with modified Rankin Scale (mRS) at admissiom and 6 months follow-up. **(A, B) **Correlation between CSF CHI3L1 levels andmRS at admissiom and 6 months follow-up in anti-LGI1 encephalitis patients. **(C, D)** Correlation between serum CHI3L1 levels andmRS at admissiom and 6 months follow-up in anti-LGI1 encephalitis patients.

The CSF CHI3L1 level were correlated with the patients’ mRS scores at admission(r =0.516, *p*= 0.004; **Figure 3A**) and 6 months later (r=0.552, *p*=0.002; **Figure 3B**). Meanwhile, serum CHI3L1 concentrations and other clinical features were not associated with the patients’ mRS score of the patients at admission (r=0.086, *p*=0.652; **Figure 3C**) and 6 months later (r=-0.003, *p*=0.988; **Figure 3D**).

The simple linear regression analysis revealed that CSF CHI3L1 levels were positively correlated with the patients’ mRS scores at admission (β=0.448, *p*=0.013), and 6-months later (β=0.450, *p*=0.013) and with the patients’ CSF lactate levels (β=0.451, *p*=0.014). The multiple linear regression analysis showed that CSF CHI3L1 levels were positively correlated with the patients’ mRS score at the 6-month follow-up examination (β=0.422, *p*=0.011) and the patients’ CSF lactate levels (β=0.420, *p*=0.012).

However, the simple linear regression analysis and multiple linear regression analysis revealed no correlation between serum CHI3L1 levels and the mRS score of the patients at onset and 6-months follow-up and other clinical features.

## Discussion

Autoimmune encephalitis is often accompanied by inflammatory cell activation and cytokineproduction during pathogenesis (15, 16). Anti-LGI1 encephalitis patients have elevated neurofilament light chain protein, glial fibrillary acidic protein and chemokine ligand 13 levels and reduced Visinin-like protein 1, Synaptosomal Associated Protein-25 (SNAP-25) and neurogranin levels in the serumand CSF (17–21). However, none of these biomarkers reflect microglia activation. CHI3L1, also has been named YKL-40 in humans, is produced by macrophages, microglial cell and neutrophils (5, 22). As a pro-inflammatory factor, CHI3L1 inhibits inflammatory cell apoptosis and death by inducing PKB/Akt phosphorylation, inhibiting Fas expression (23). Moreover, CHI3L1 promotes the activation and differentiation of immune cells, such as macrophages, dendritic cells and T lymphocytes (24).

Some studies have indicated that elevated CSF CHI3L1 are associated with Alzheimer’s disease, multiple sclerosis and Parkinson’s disease (22, 25–29). One study has shown that CHI3L1 levels are significantly elevated in the CSF of patients with anti-LGI1 encephalitis. However, that study only included seven patients (21) Patients with anti-NMDAR encephalitis also have elevated CSF CHI3L1 levels (11, 12). Additionally, CSF CHI3L1 levels are associated with the mRS score both at admission and 6 months later (11, 12, 16) An [18F]-DPA714 PET/CT scan study revealed that one patient with recurrent anti-LGI1 encephalitis had activated microglia in the left medial temporal lobe indicating that microglia play a major role in the pathogenesis of anti-LGI1 encephalitis (30). Active microglia might increase CHI3L1 levels in the CSF. Furthermore, CHI3L1 increases T lymphocyte levels, particularly Th2 cells in type 2 inflammatory responses (31). Additionally, neutrophils and endothelial cells also secrete CHI3L1 and may, therefore, contribute to the elevation of CSF CHI3L1 levels in LGI1 patients (32, 33).

The present study, that patients with anti-LGI1 encephalitis had elevated serum and CSF CHI3L1 levels. CSF CHI3L1 levels were correlated with mRS scores at admission and 6 months later. Moreover, CSF CHI3L1 levels were higher in anti-LGI1 encephalitis patients presenting with cognitive impairment than those without cognitive impairment symptoms. Note-worthily, patients presenting with only FBDS at admission had significantly lower CSF CHI3L1 levels than patients with other symptoms.

These results imply that CSF CHI3L1 levels were associated with severity and outcomes. Most patients presenting with only FBDS at admission did not suffer from serious anti-LGI1 encephalitis. A recent Mayo Clinic study have reported that more than half of patients with anti-LGI1 encephalitis presented with FBDS (34). Those patients with FBDS have no obvious inflammatory responses in the CSF at the early disease stage (35). In these patients, early immunotherapy usually yields good results (36, 37).

Our results revealed that the mRS score at admission was positively correlated with hyponatremia, suggesting that hyponatremia is also related to the severity of the disease. One study demonstrated that the prognosis of anti-LGI1 encephalitis patients with hyponatremia is poor (38). In this study, CSF CHI3L1 levels were correlated with CSF lactate levels. These results suggest that metabolic and inflammatory factors are both involved in the pathogenesis of patients with anti-LGI1 encephalitis.

There are some limitations to our study. First, the patients population remains relatively small. Second, the serum and CSF samples obtained in the follow-up examination were not studied.

## Conclusion

CSF CHI3L1 levels are correlated with the severity and prognosis of anti-LGI1 encephalitis.

## Data availability statement

The raw data supporting the conclusions of this article will be made available by the authors, without undue reservation.

## Ethics statement

The studies involving human participants were reviewed and approved by ethics committee of the Qilu Hospital of Shandong University. The patients/participants provided their written informed consent to participate in this study.

## Author contributions

JL and HL, drafting/revision of the manuscript, data acquisition, study concept and design, and data analysis and interpretation. YW, major role in sample collection. XZ, SW and LL, revision of the manuscript, study concept and design, and data analysis and interpretation. All authors contributed to the article and approved the submitted version.
